# Hearing and music in unilateral spatial neglect neuro-rehabilitation

**DOI:** 10.3389/fpsyg.2014.01503

**Published:** 2014-12-23

**Authors:** Alma Guilbert, Sylvain Clément, Christine Moroni

**Affiliations:** Equipe Neuropsychologie et Cognition Auditive, Laboratoire de Neurosciences Fonctionnelles et Pathologiques-EA 4559, UFR de Psychologie, Université Charles de Gaulle, Lille 3, Villeneuve d’Ascq, France

**Keywords:** unilateral spatial neglect, rehabilitation, sensory retraining, hearing, music

## Abstract

Unilateral spatial neglect (USN) is an attention deficit in the contralesional side of space which occurs after a cerebral stroke, mainly located in the right hemisphere. USN patients are disabled in all daily activities. USN is an important negative prognostic factor of functional recovery and of socio-professional reinsertion. Thus, patient rehabilitation is a major challenge. As this deficit has been described in many sensory modalities (including hearing), many sensory and poly-sensory rehabilitation methods have been proposed to USN patients. They are mainly based on visual, tactile modalities and on motor abilities. However, these methods appear to be quite task-specific and difficult to transfer to functional activities. Very few studies have focused on the hearing modality and even fewer studies have been conducted in music as a way of improving spatial attention. Therefore, more research on such retraining needs is neccessary in order to make reliable conclusions on its efficiency in long-term rehabilitation. Nevertheless, some evidence suggests that music could be a promising tool to enhance spatial attention and to rehabilitate USN patients. In fact, music is a material closely linked to space, involving common anatomical and functional networks. The present paper aims firstly at briefly reviewing the different procedures of sensory retraining proposed in USN, including auditory retraining, and their limits. Secondly, it aims to present the recent scientific evidence that makes music a good candidate for USN patients’ neuro-rehabilitation.

## INTRODUCTION

Unilateral spatial neglect (USN) is a neuropsychological syndrome characterized by an attention deficit in the contralesional side of space (Posner et al., 1984). USN cannot be linked to a sensory or a motor deficit. USN patients fail to orient themselves toward contralesional targets. Many spatial deficits can appear with USN patients: colliding with left side objects while walking, dressing only one side of their body or failing to eat the food on the neglected side of their plate. Thus, they are severely disabled in all daily activities. This syndrome results mainly from a right hemispheric damage after a stroke (Bartolomeo et al., 2012). Cerebral lesions could be located in a large territory in the brain, going from the parietal lobe to the frontal one. Stone et al. (1991) found that 72% of patients with a right cerebral stroke had USN 3 days after. After 3 months, 33% of these patients still showed signs of neglect signs which tended to last for years. This deficit is an important negative prognostic factor of functional recovery (Held et al., 1975; Denes et al., 1982). Therefore, its rehabilitation is a major challenge as USN could negatively influence motor recovery and also social and professional reintegration.

A variety of clinical tests exist to assess USN. Assessment usually relies on “paper and pencil” tests. The most common conventional tests are the line bisection tasks (Halligan and Marshall, 1991), the cancelation tasks, such as the star cancelation task (Wilson et al., 1987) and the bells test (Gauthier et al., 1989). Simple drawing, copying of figures, reading and writing tasks are also used. In each of them, the number of reported elements located in the contralesional side of space is considered in order to evaluate the presence of USN. However, these tasks have several limitations. For example, they are essentially based on visual modality and they do not identify the daily life difficulties of patients. Some batteries of tests have been designed in this aim. The Behavioural Inattention Test (BIT; Wilson et al., 1987) is the most commonly used test and consists of both the aforementioned “paper and pencil” tests and behavioral procedures to evaluate USN. However, its ecological validity remains questionable: behavioral procedures are not more sensitive than conventional tests to detect USN (Halligan et al., 1991). Therefore, an evaluation of daily life activities appears to be necessary to evaluate the functional impact of USN. However, only a very few scales exist, such as the Catherine Bergego Scale (Bergego et al., 1995; Azouvi et al., 2003). Due to the lack of assessment tools, this aspect is often forgotten during clinical assessments.

Although USN is essentially evaluated in the visual modality in clinical practice, deficits of spatial attention in USN have been described in many other sensory modalities: touch (De Renzi et al., 1970; Barbieri and De Renzi, 1989), hearing (De Renzi et al., 1989; Pavani et al., 2001), and proprioception (Vallar et al., 1993). Barbieri and De Renzi (1989) characterized tactile neglect as a failure to detect tactile stimulations on the left side of a patient’s body. In hearing, USN patients may show errors in localizing and lateralizing sounds (Pavani et al., 2001) often shifting them toward the right side. In addition, they may present real auditory neglect with a failure to detect a stimulus perceived with the left ear (De Renzi et al., 1989). Vallar et al. (1993) also showed that USN patients have a deficit in their position sense when compared to right damaged patients without USN signs. In other words, USN patients have difficulties in localizing themselves in space. Moreover, in USN, motor neglect with a spontaneous under-use of the contralesional arm could also be observed without hemiplegia or other deficits (Laplane and Degos, 1983; Coulthard et al., 2008).

Even though there are only a limited number of studies on USN in modalities other than the visual modality (particularly because of the lack of clinical tools to assess them), USN seems to affect all sensory modalities and not only vision. De Renzi et al. (1970) suggest that USN is caused by mutilated space representation and this can affect all the sensory modalities. Therefore, USN can be interpreted as a supramodal spatial bias even though some differences of severity between modalities can be found (Chokron et al., 2002).

In the next section, we will conduct a review of the main sensory rehabilitation methods based on the theoretical conception that USN is a spatial attention deficit involving different sensory modalities. In the following sections, after underlying the contribution of hearing in USN rehabilitation, we will present the recent scientific evidence that makes music and musical practice promising tools for USN patients’ rehabilitation.

## SENSORY RETRAINING

The visual scanning training method (Diller and Weinberg, 1977; Pizzamiglio et al., 1992) is the most commonly used method in clinical practice. The underlying theory of this method is that USN is a spatial exploration deficit and retraining this top-down processing can lead to rehabilitation. Patients learn to voluntarily pay attention to the left side of visual space due to an initial target placed on their left side. This target is used as a visual anchoring point and its salience decreases session after session so that USN patients have to pay more and more attention by themselves. Various tasks such as reading tasks or cancelation tasks can be used with this method. The complexity of these tasks can also be enhanced during training sessions by increasing the number of distractors in the cancelation task or reducing the letter size of the text in the reading task. This rehabilitation provided quite good results on USN neuropsychological tests (Diller and Weinberg, 1977; Pizzamiglio et al., 1992). However, this rehabilitation used similar tasks to those included in the neuropsychological assessment, therefore preventing a generalization of the training effects in everyday life (Seron et al., 1989; Wagenaar et al., 1992). The gains due to this retraining appear to be task-specific and not transferable to functional activities.

Other rehabilitation methods exist and are based on the hypothesis that USN is an impairment of coordinate transformation used to represent extra-personal space and can be rehabilitated by recalibrating the perception of space. These rehabilitation techniques aim at modifying cognitive maps to induce more accurate behaviors. The cases of optokinetic stimulations or prismatic glasses in the visual modality are examples of such techniques. In other modalities, caloric or transcutaneous electrical stimulations, also rely on this hypothesis. Optokinetic stimulations are based on displays of visual stimuli all moving coherently on a computer screen to the patient’s neglected side. Some studies (Vallar et al., 1993; Karnath, 1996) showed improvements in visual scanning in the neglect field and an improvement in the deficit in their position sense with this method. According to Kerkhoff et al. (2006), the presentation of moving visual stimuli with active smooth pursuit eye movement can be more efficient than the conventional training of visual scanning. Finally, in a study by Pizzamiglio et al. (2004), one group of patients received only visual scanning tasks as a form of rehabilitation whereas another group received visual scanning tasks combined with optokinetic stimuli. This latter situation did not show more efficiency than visual scanning using only static stimuli. Moreover, these studies did not evaluate the potential effects on daily life. Prismatic glasses, which modify the perception of visual environment so as to induce a gaze deviation to the left, are another method to rehabilitate USN patients (Rosetti et al., 1998; Serino et al., 2009). These prismatic glasses generally deviate the visual field 10° to the right. Unlike in the visual scanning training method, the prismatic glasses modify perceptual environment in order to change cognitive maps. They use bottom-up processing to rehabilitate USN. In this treatment, patients wear prismatic glasses and have to go through target-pointing tasks. This device has shown promising results. Unfortunately, these results dure often only in the short-term [in the study by Rosetti et al. (1998) the results dured almost 2 h]. Prismatic glasses involve a habituation mechanism to induce a change in the cognitive maps. When the patient removes the glasses, the spatial habituation continues for a certain time. However, the visual perception without glasses will involve a new change in cognitive maps with a return to the previous and pathological ones. This explains the short-term efficiency of this treatment. Furthermore, some studies (Rousseaux et al., 2006) did not find any effect (for a review, see Rode et al., 2006).

Other rehabilitation methods using the vestibular and the somato-sensory modalities have also been used when treating USN. These methods are based on the same hypothesis of the two previous techniques and aim to calibrate cognitive maps. Vestibular stimulation, also known as caloric stimulation, involves the use of cold water in the left ear (Rode et al., 1998). In this method, caloric stimulation has been found to reduce USN symptoms. However, this remission is temporary, only lasting from thirty to sixty minutes. Transcutaneous electrical stimulation can also be used. Vallar et al. (1995) showed that a left neck electrical stimulation could improve performance on line or letter cancelation tests in 13 of the 14 USN patients included in their study. These two previous methods are efficient in treating USN symptoms. However, the major issues are similar to those faced with in visual scanning training methods and concern generalization to daily life and long-term efficiency. These methods yield important but temporary remission of deficits.

Other approaches target motor abilities in order to help the rehabilitation of visual attention. Using motor rehabilitation (Robertson et al., 1992), it was shown that a contralesional arm mobilization could reduce USN signs for several weeks after treatment. Robertson and North (1992) underlined that a contralesional arm activation in the contralesional hemi-space was more successful in reducing USN signs on a cancelation task than one in the other hemi-space.

Finally, many rehabilitation techniques have been proposed on sensory modalities other than the visual one but difficulties in evaluating the impact of these techniqus were reported as we have previously seen. Indeed, the clinical tools used to evaluate the efficiency of a rehabilitation method are essentially based on the visual modality, and overall, do not take into account the possible improvement in other modalities and, rarely, in daily life. In order to come closer to real life improvements, studies using poly-sensory rehabilitation methods have emerged.

## POLY-SENSORY RETRAINING

Since we live in a multi-sensory environment, combining modalities bring the rehabilitation closer to real life. Brunila et al. (2002) chose to use motor skills with left arm activation combined with visual scanning training in order to help the latter and showed considerable improvement on cancelation tasks. Nevertheless, there was no improvement found in other tasks. In their design, only one rehabilitation patient group was used. This group participated in a training program which combined left arm activation with visual scanning. The authors concluded that the gain effect could have been caused by an efficient combination of the two methods, visual scanning training and left arm activation. However, the observed effect also could have been caused by the efficiency of only one of the two previous methods. This study could not dissociate the two hypotheses as no control group was used. In order to answer this question, Luukainen-Markkula et al. (2009) compared the visual scanning training method to the left arm motor activation rehabilitation method. They underlined that the efficiency of the two programs were roughly the same and that both could have been useful in the approach of Brunila et al. (2002). These authors used this finding to argue that combine modalities and efficient rehabilitation methods could be a better way to rehabilitate USN.

Recently, Polanowska et al. (2009) combined a left-hand somato-sensory electrical stimulation with the visual scanning training method. Their study demonstrated that patients who received the combination technique had better visual exploration results for than those who received only the visual scanning training method. The authors argued that the electrical stimulations involved an activation of the attention system of the right cerebral hemisphere and would help visual exploration. Therefore, multi-sensory retraining appears necessary. However, there was no ecological test and no functional evaluation, except the Barthel Index (Mahoney and Barthel, 1965). This index is not specific to USN patients but was created to evaluate physical disabilities and did not show any improvement in this study. Difficulties for daily life generalization can still persist, as the previous methods showed, which are difficult to underline by the tests used in a clinical approach. Developing and using more ecological tests appears necessary to evaluate the daily life use of the poly-sensory retraining program, even though, it provides better results than retraining based on only one sensory modality. Finally, it is important to highlight that no rehabilitation based on more than two sensory modalities was found in the literature and that poly-sensory retraining always involves the visual modality. The improvement of the other modalities after rehabilitation is often under-assessed and these modalities are also less used in USN rehabilitation.

## CONTRIBUTION OF HEARING IN RETRAINING

And what about hearing? In literature, very few studies take an interest in the potential effects of auditive stimulations in USN rehabilitation. This is probably due to a lack of audition assessment tools. However, some studies have provided evidence that auditory stimuli could significantly enhance visual perception in USN. These patients show an improvement in visual detection when visual and auditory stimuli come from the same position in space, unlike when they came from two different positions (Frassinetti et al., 2002a,b). This improvement is greater for visual positions affected by the USN, that is to say in the left part of space most of the time. These studies have underlined the existence of an integrated visuo-auditory system in USN patients and the importance of using auditory stimulations in order to help visual detection.

Furthermore, some evidence of auditory stimulations as an effective modality training for USN patients exists. Hommel et al. (1990) proposed that, in USN patients, music stimuli could be superior to other sensory or cognitive cues (such as speech or tactile cues). In their study, listening to binaural non-verbal auditory stimuli decreases USN symptoms. Patients reported more elements in the left side in a copying drawings task, whereas binaural auditory verbal stimuli and unilateral or bilateral tactile stimuli have no effect on this task. This effect was found with classical music such as with white noise, suggesting that this effect is not specific to music. The effect can be linked to a greater activation in the right cerebral hemisphere compared to the left one, whereas it was not the case with verbal stimuli. According to the authors, verbal stimuli imply a bilateral hemispheric activation and a persistence of interhemispheric imbalance in USN patients. These results underlined that auditory cerebral pathways could take part in the network connected to USN and non-verbal passive auditory stimuli could improve USN symptoms. Therefore, this could have a real relevance in regards to circumvent visual modality as deficits could be major in vision and hearing seems to be a valid alternative.

Nevertheless, contrary to all other sensory modalities, no study was really focused on the specific effect of the auditory stimulations in long-term rehabilitation. Some studies used auditory stimulations combined with other sensory rehabilitation methods. Yet, in these rehabilitation methods, auditory stimuli were only used as feed-back information or as alerts but not as a real rehabilitation tool (Fanthome et al., 1995; Robertson et al., 1998) with an analysis of the specific effects of auditory stimulations on USN symptoms.

## MUSIC IN USN REHABILITATION

Music as an auditory stimulation is particularly interesting to examine. This is because, as we see in Hommel et al.’s (1990) study, music activates more the right cerebral hemisphere than the left one and, therefore, could improve neglect.

Recently, studies showed that just listening to music could improve spatial attention in USN (Soto et al., 2009; Chen et al., 2013; Tsai et al., 2013). The visual awareness on the contralesional hemi-space increased when visual tasks were performed with patient’s preferred music relative to their un-preferred music or silence (Soto et al., 2009). Preferred music enhanced the detection and the identification of contralesional targets in a perceptual report task, enabled patients to make more accurate midline bisection judgments and improved the reading on the controlesional side of space. However, only three patients were included in this study. Chen et al. (2013) did a similar experiment on nineteen patients and also established that listening to pleasant music has a positive impact on three subtests in the BIT (the Star Cancelation Test, the Line Bisection and the Picture Scanning Test) and, so may improve visual attention in USN patients. Similarly, results in Tsai et al. (2013) indicated that listening to a classical music increases performances on the same three subtests of the BIT. Music appears to be a promising tool to rehabilitate USN.

Even with the possibilities presented, music as auditory stimulations has not yet been used as tool for long-term rehabilitation in USN. From our review of the literature, every few studies using music material to rehabilitate USN patients was found. However, music has more frequently been used in long-term rehabilitation of patients with a cerebral stroke. Sarkamo et al. (2008) conducted a study with 55 patients with a left or right hemisphere middle cerebral artery stroke in which patients were assigned to a music group, a language group or a control group. Every day for 2 months, the music and the language groups listened to self-selected music or to audio-books, whereas the control group did not receive any listening material. Results showed that self-selected music listening during the early post-stroke stage could enhance verbal memory and focused attention even more than listening to audio-books. Verbal memory was evaluated thanks to a story recall subtest and a list-learning test whereas focused attention was assessed with mental subtractions and the Stroop test. No significant effect was found on the other cognitive domains. Furthermore, listening to music was indicated to prevent negative moods. In this study, 16 USN patients were included. However, their results were not analyzed separately from the other patients. In addition, the influence of music on spatial attention, and, especially on USN, was not precisely studied in this paper. How could music have a greater influence on cognition than stories? It is important to underline that some musics with lyrics were used in this study. Therefore, this material can provide more activation than the stories because the stories activate essentially the left cerebral hemisphere more dedicated to language whereas the combination of music and language can activate both cerebral hemispheres (Callan et al., 2006). Consequently, this can have a greater impact on cognition. Additionally, in this study, music was shown to prevent depression. This suggests that music is closely linked to mood and emotions.

## MUSIC EFFECT, AN EFFECT OF POSITIVE EMOTIONS INDUCED?

The question can arise regarding how music could enhance spatial attention in USN. Could the effects of music be specific to space or be linked to more general non-spatial effects, such as those induced by emotions?

The “Mozart effect” was the first piece of evidence in favor of interactions between music and space (Rauscher et al., 1993). This effect suggests that listening to Mozart’s music can enhance visuo-spatial abilities. In their experiment, the authors found that a visuo-spatial quotient, calculated with series of tests assessing intellectual quotient, was higher for college students, just after listening to a Mozart’s sonate. Rauscher et al. (1995) assumed that the same cerebral areas were activated by listening to a Mozart’s sonate and when performing visuo-spatial tasks. However, this effect was not replicated by some studies (Steele et al., 1999). Moreover, Mehr et al. (2013) showed no cognitive effects of music instruction in children, suggesting that the “Mozart effect” is a temporary effect. Thus, a number of authors underlined that the “Mozart effect” could essentially be caused by a mood factor provoked by high tempo music (Thompson et al., 2001; Husain et al., 2002). This interpretation involving the influence of positive emotions induced by music has also been evoked by Soto et al. (2009).

Listening to pleasant music can influence emotional states and activate the substrates of emotional states in the orbito-frontal cortex (Dellacherie et al., 2009). According to Salimpoor et al. (2011), pleasant music produces a dopamine release in the mesocortico limbic reward system, precisely in the ventral and dorsal striatum. This increase of dopamine is at the origin of the activation of the orbito-frontal cortex. It also directly has an impact on other brain regions, such as ventral medial prefrontal cortex or hippocampus. Therefore, this dopamine release can enhance global cognitive functioning in patients with cognitive deficits (Nagaraja and Jayashree, 2001) as well as alertness, speed of information and memory in healthy individuals (Schuck et al., 2002).

Soto et al. (2009) hypothesized that these activated emotional regions could modulate the activity of intact attentional brain areas located in the intra-parietal cortex and, thus, increase visual awareness and, by the way, spatial attention.

Are these effects specific to music? Thompson et al. (2001) proposed the “arousal and mood hypothesis” that explains how cognition can be influenced by music. This hypothesis states that any enjoyable stimuli can induce positive affect and increase arousal and, consequently, improve cognitive performances. According to this hypothesis, this positive effect is not specific to music, it is essentially provoked by the emotional aspect of music. This interpretation was used by Sarkamo et al. (2008). Nevertheless, in neglect patients, another hypothesis could be assumed to explain why music could be useful to rehabilitate spatial attention. This hypothesis is based on more specific links between music and space and will be discussed in the next section.

## LINKS BETWEEN SPACE AND MUSIC

Music could have a specific effect on the spatial attention of USN patients, not only induced by positive emotions. Space and music have close links. Some studies, carried out with healthy participants, showed that musicians had, on average, better visuo-spatial abilities than non-musicians. These studies have emphasized the idea of direct interactions between music and space. For example, the study by Brochard et al. (2004) indicated better performances in a perceptual and a mental visual imagery task in musicians than in non-musicians. Musicians were faster to associate a visual stimulus with a specific motor response. The authors assumed that daily practice of a musical instrument could have greatly improved basic perceptuomotor abilities, by reorganizing some cerebral networks.

The same cerebral areas, located in the parietal cortex are activated by tasks comparing different musical pitches and visuo-spatial tasks. Foster and Zatorre (2010) demonstrated the implication of the intra-parietal sulcus (IPS) in transforming musical pitch information. It should be noted that the IPS has a major role in space perception and in USN syndrome (Corbetta et al., 2005). Corbetta et al. (2000) also showed a hypo-activation of the IPS during spatial attention orienting task in all of their USN patients, indicating that there are close links between cerebral networks implicated in space attention and in music. Could music be a way to re-activate, or even reorganize, this specific network in the brain?

We can question why listening to musical tones can activate cerebral areas also related to space. The main considered hypothesis is that pitches can be conceptualized as positions on a mental line oriented either vertically or horizontally (Rusconi et al., 2006; Lidji et al., 2007). This hypothesis is inspired by the theory of the mental number line in which numbers are represented in a continuous analogical format (Dehaene, 2001). This means that numbers are ranked according to their magnitude on a mental line from left to right.

Zorzi et al. (2002) pointed out the fact that this mental number line could be disrupted in neglect patients. In this study, when USN patients had to estimate the midpoint of a numerical interval, a right-deviation in their answers was observed. These errors did not look like an acalculia but referred to their errors in bisecting lines. In addition, patients with right brain damage but without USN did not show this error pattern in this task. These findings suggested that numbers could have spatial representations.

According to some authors, numbers are not the only items linked to space. Walsh (2003) proposed A Theory Of Magnitude (ATOM) which suggests that space, numbers and other magnitudes are located in the inferior parietal cortex and share common representations. Bueti and Walsh (2009) have proposed that areas implicated by different sources of magnitude information are in the same cerebral region in order to improve the sensory-motor performance.

Lidji et al. (2007) carried out a series of experiments to prove the hypothesis of a mental pitch line. They showed using a Spatial Pitch Association Response Codes (SPARC) effect that a vertical line was activated automatically either in a pitch association task or in an instrumental timbre judgment task. This pattern of results was observed in both musician and non-musician participants. In fact, if two buttons were vertically displayed, participants were faster to associate low-pitched tones with the bottom button and high-pitched tones with the top one. This vertical mapping of pitch is congruous with adjectives “low” and “high” used to describe the pitch of auditory stimuli. People associate pitch height with a spatial height.

The same authors also demonstrated the existence of a horizontal mental pitch line. The results were influenced by musical expertise of participants. Musicians seemed to associate automatically low-pitched tones with the left part of a horizontal line and high-pitched tones with the right part. For non-musicians, this association was found only with an explicit pitch comparison task and not with an instrumental timbre judgment task. These findings are congruent with those found by Rusconi et al. (2006) who carried out similar experiments on the SPARC effect.

Finally, Lidji et al. (2007) tested if an ascending or a descending melodic interval could activate either a left-to-right or a bottom-top association between spatial position and the orienting of the melodic interval change. No significant Spatial Melody Association Response Codes (SMARC) effect was found in this experiment in non-musicians. Participants were not faster to associate a descending melody with a left or bottom button and an ascending melody with a right or top button. Thus, melody was found to be too complex to be treated as spatial material. Overall, these findings indicate clearly that spatial areas are activated by a task using a pitch judgment and therefore could be activated by listening to music.

As USN patients could show impairment in the estimation of a number interval midpoint, we can ask ourselves if estimation of all magnitudes, and especially of pitches, could be affected by USN. Cusack et al. (2000) showed impairments in a pitch discrimination task in USN. Their patients had difficulties in localizing a sound relative to another in terms of frequency whereas no difficulty was found in one-interval task. These patients were able to say if a single sound was modulated in frequency. The authors concluded that their USN patients had a specific auditory deficit between-object comparisons and that within-object comparisons were entirely un-impaired. This study could be linked to those conducted by Lidji et al. (2007). USN patients have impairment in spatial cerebral areas, these impairments could affect the mental pitch line. The fact that one-interval tasks were well-performed could be compared with the fact that ascending or descending melodies do not imply a mental line. These tasks may involve other cerebral areas not impaired in USN.

Pavani et al. (2002) did not show difficulty in a pitch discrimination task. In this study, USN patients succeeded in discriminating a lower tone from a higher one similarly as a control group of patients with a right hemispheric lesion without USN. However, in this study, just two pitches were used, making the task too easy and not sensitive enough to detect a difficulty. Furthermore, USN patients had to categorize the sound they heard as “high” or “low” rather than localizing a pitch tone relative to another. This task could not imply spatial pitch representation. Therefore, pitch discrimination ability needs to be explored in USN with complementary studies.

How could these findings help to rehabilitate USN patients? Recently, Ishihara et al. (2013) have shown that listening to higher or lower pitches can modulate a line bisection task, either for healthy individuals or for right brain damaged patients with or without USN. A lower pitch induces a leftward or downward bias whereas a higher pitch involves a rightward or upward deviation. These effects were identified as greater only for the unique USN patient included in their study. In this patient, the bisection performance gradually increased during the experiment. Although only twenty lines were used in the experimental situation, this patient still showed an improvement on this task 1 week later. This study suggests that non-lateralized auditory cues, especially pitches, could influence the direction of the attentional bias in USN. The authors have underlined the fact that these auditory cues could be used as a long-lasting rehabilitative treatment. Thus, one more time, more researches are needed to assess the efficiency of using pitches as spatial cues in a USN rehabilitation.

Although pitch discrimination has not been studied enough in USN, evidence seems to show that music could activate spatial representations in the brain and, therefore, could be a successful tool to use in the rehabilitatation of USN.

## MUSICAL PRACTICE: A PROMISING TOOL TO REHABILITATE USN?

Musical practice appears to be a promising tool to rehabilitate USN for several reasons. First, playing music involves several sensory modalities: hearing, vision, touch and motor skills. Musical practice also implies higher-order cognitive processes. Musicians learn associations between motor actions, specific sound and visual stimuli and receive, in return, multisensory feedback. According to Wan and Schlaug (2010), these associations can strengthen connections between auditory and motor regions and activate multimodal integrations regions around the IPS, which is hypo-activated in USN (Corbetta et al., 2000). In addition, as mentioned earlier, the fact that music could activate spatial representations in brain has been stressed either for musicians or for non-musicians (Lidji et al., 2007). Therefore, playing music could be an interesting way to re-activate brain networks impacted by USN.

Finally, playing music could be have a greater impact than other methods. USN patients can have difficulties in being implicated in their own rehabilitation, in particular because of anosognosia (i.e., unawareness of the neurological deficit). Appelros et al. (2007) estimated that anosognosia touched 43% of USN patients and partly explained why rehabilitation fails for some patients. Musical practice may be more pleasant than commonly used rehabilitation methods and, therefore, could be less challenging for patients who are not aware of their difficulties.

The real efficiency of musical practice has not been sufficiently examined in USN. In the literature, just one study, in which only two USN patients were included, implied musical practice (Bodak et al., 2014). The authors showed that an active period of music-making with a horizontally aligned instrument (chime bars) could reduce attentional bias. Unfortunately, only conventional tasks were used to evaluate USN in this study (cancelation tasks, line bisection,…) and no ecological test was included and, therefore, we cannot conclude on potential daily life generalization. Furthermore, this study did not include another rehabilitation method to compare the efficiency of musical practice and so the observed improvement could only be an effect due to the introduction of a retraining and not a specific effect as it was expected to prove. Clearly, more research is necessary in order to fill the theorical and applicative gaps found in the literature concerning music and, more particularly, musical practice.

## CONCLUSION

Several sensory rehabilitation techniques have been proposed for USN patients. As we saw in this review, a number of them focus only on the visual modality whereas others use motor skills, somato-sensory or vestibular stimulations to improve visual spatial attention. These rehabilitation methods are mainly based on two theoretical approaches: either recalibrating cognitive maps or retraining the orientation of spatial attention. The literature indicates that poly-sensory rehabilitation appears to be a better way to rehabilitate USN. However, all these methods present major issues concerning the generalization to daily life and the long-term efficiency.

In the light of this review, both music listening (perception) and music playing (production) have been indicated as promising methods to rehabilitate spatial attention in USN patients. Music involves general effects on cognition linked to motivation and the emotions that it induces (the heightened arousal produced by music has an impact on cognition). We have seen that music could contribute more specifically to the orientation of spatial attention as music and space share closed links. Notably, musical practice could be an interesting rehabilitation tool as it also involves several sensory modalities (hearing, vision, arm movements, etc). It is essential that more research is conducted on music and musical practice in order to determine their potential effects on spatial attention in USN patients.

### Conflict of Interest Statement

The authors declare that the research was conducted in the absence of any commercial or financial relationships that could be construed as a potential conflict of interest.
